# Operational description of rare diseases: a reference to improve the recognition and visibility of rare diseases

**DOI:** 10.1186/s13023-024-03322-7

**Published:** 2024-09-11

**Authors:** Chiuhui Mary Wang, Amy Heagle Whiting, Ana Rath, Roberta Anido, Diego Ardigò, Gareth Baynam, Hugh Dawkins, Ada Hamosh, Yann Le Cam, Helen Malherbe, Caron M. Molster, Lucia Monaco, Carmencita D. Padilla, Anne R. Pariser, Peter N. Robinson, Charlotte Rodwell, Franz Schaefer, Stefanie Weber, Flaminia Macchia

**Affiliations:** 1 Rare Diseases International, Plateforme Maladies Rares, 96 rue Didot, Paris, 75014 France; 2 INSERM, US14 - Orphanet, Plateforme Maladies Rares, 96 rue Didot, Paris, 75014 France; 3 Federación Argentina de Enfermedades Poco Frecuentes, Antonino Ferrari 1044, 1424 Buenos Aires, Argentina; 4 Chiesi Farmaceutici SpA, Via Paradigna 131/A, Parma, Italy; 5 Rare Care Centre, Western Australian Department of Health, Perth Children’s Hospital and Western Australian Register of Developmental Anomalies, King Edward Memorial Hospital, Subiaco, WA Australia; 6 School of Medicine, The University of Notre Dame Australia (UNDA), 160 Oxford St, Darlinghurst, NSW Australia; 7 Johns Hopkins University School of Medicine, 600 N. Wolfe Street, Blalock 1008, Baltimore, MD 21287-4922 USA; 8 EURORDIS - Rare Diseases Europe, Plateforme Maladies Rares, 96 rue Didot, Paris, 75014 France; 9 Rare Diseases South Africa, 63 Peter Place, Bryanston, Sandton, Johannesburg, South Africa; 10 Office of Population Health Genomics, West Australia Department of Health, Level 3, C Block, 189 Royal Street, East Perth, WA, 6004 Australia; 11 International Rare Disease Research Consortium (IRDiRC), Paris, France; 12 Department of Pediatrics, College of Medicine, University of the Philippines Manila, Pedro Gil Street, Taft Ave, Ermita, Manila 1000 Philippines; 13 The Jackson Laboratory for Genomic Medicine, 10 Discovery Drive, Farmington, CT 06032 USA; 14 European Rare Kidney Disease Reference Network (ERKNet), Medical Faculty Heidelberg, Heidelberg University, Im Neuenheimer Feld 130.3, 69120 Heidelberg, Germany; 15 Department K - Code Systems and Registers, Federal Institute for Drugs and Medical Devices, Kurt-Georg- Kiesinger-Allee 3, D-53175 Bonn, Germany; 16 Centre for Human Metabolomics, North-West University, Potchefstroom, South Africa

**Keywords:** Rare diseases, Visibility, Definition, Coding, Healthcare systems, Prevalence, Diagnosis, Social care, Research and development, Universal health coverage

## Abstract

Improving health and social equity for persons living with a rare disease (PLWRD) is increasingly recognized as a global policy priority. However, there is currently no international alignment on how to define and describe rare diseases. A global reference is needed to establish a mutual understanding to inform a wide range of stakeholders for actions. A multi-stakeholder, global panel of rare disease experts, came together and developed an Operational Description of Rare Diseases. This reference describes which diseases are considered rare, how many persons are affected and why the rare disease population demands specific attention. The operational description of rare diseases is framed in two parts: a core definition of rare diseases, complemented by a descriptive framework of rare diseases. The core definition includes parameters that permit the identification of which diseases are considered rare, and how many persons are affected. The descriptive framework elaborates on the impact and burden of rare diseases on patients, their caregivers and families, healthcare systems, and society overall. The Operational Description of Rare Diseases establishes a common point of reference for decision-makers across the world who strive to understand and address the unmet needs of persons living with a rare disease. Adoption of this reference is essential to improving the visibility of rare conditions in health systems across the world. Greater recognition of the burden of rare diseases will motivate new actions and policies to address the unmet needs of the rare disease community.

**Supplementary Information:**

The online version contains supplementary material available at 10.1186/s13023-024-03322-7.

## Introduction

Improving health and social equity for persons living with a rare disease (PLWRD) and their families, is increasingly recognized as a global policy priority. Rare diseases are recognized within the United Nations (UN) 2030 Agenda for Sustainable Development, which seeks to promote equity by addressing the needs of the most vulnerable populations. In 2019, countries adopted the UN Political Declaration on Universal Health Coverage that includes mention of rare diseases [1]. In 2021, the UN General Assembly adopted the UN Resolution: “Addressing the Challenges of Persons Living with a Rare Disease and their Families” [2]. As public health agencies and governments implement new policies, they need a reference that describes *which* diseases are considered rare, *how many* persons are affected, and *why* the rare disease community deserves specific attention [3]. There is currently no collectively agreed definition for rare diseases that integrates these three elements, to recognize the distinct challenges, burden, and impact of these conditions on PLWRD, their families, health systems, and society. While many countries have developed or adopted a definition of rare diseases, they vary across different countries and regions [3–7]. Most existing definitions are based on prevalence or historically were set up to provide incentives for drug development. However, most rare diseases do not have, and may never have, treatments; consequently, it is necessary to look beyond a definition that was designed for regulatory purposes. An internationally recognized and comprehensive description of rare diseases will allow stakeholders to gather and collaborate on a common understanding to improve on the lives of PLWRD and their families. A global reference will facilitate data collection and comparisons across countries, identify PLWRDs for inclusion in clinical research and advance therapy development. The enhanced data collection and knowledge generation will support better care coordination and strengthen health care systems to provide services to PLWRDs. An internationally endorsed description of rare disease will inform Member States on what is at stake, support the implementation of the UN Resolution to address challenges at all levels, and support collaboration among international organizations and agencies.

A multi-stakeholder panel of experts gathered in 2022 and developed an internationally endorsed reference, the Operational Description of Rare Diseases, presented in this position statement article. The participants in the expert group included clinicians, researchers, terminology producers, medical statisticians, industry expert, patient representatives, policy makers and public health experts. Experts took part in three online workshops and two surveys, and used the online modified-Delphi process to build consensus (see Supplementary Material). The methodology focused on building consensus among the experts and is not meant as a scientific research. The resulting Operational Description of Rare Diseases is presented in this position statement. It establishes a common point of reference to describe rare diseases, to inform decision-makers and as a baseline to support future discussions. Adoption of this reference is essential to improving the visibility of all rare conditions in health systems at local, national, and international levels. Recognition of the burden of rare diseases will allow for new actions and policies to address the needs of PLWRD.

### Concepts underlying rare and disease

To develop a consensus on what is a rare disease, it is necessary to define what is “rare” and what is “disease” (Table 1).

Rarity is often described by an epidemiological threshold to recognize persons living with conditions that are of low prevalence or incidence in the general population. Existing rare disease definitions differ across countries and regions [5]. Prevalence thresholds are often set to drive policy responses according to specific unmet needs. For example, minimizing disparity in indigenous populations and ethnic minority groups, boosting research and development, or national planning [6, 8–11]. In addition, prevalence of rare diseases may vary across regions due to different demographics, founder, or environmental factors. Although countries may use a threshold based on their distinct needs, convergence towards a standard applied within a larger geography, for instance the World Health Organization (WHO) defined regions, should be encouraged.

Different approaches are being used to define rare diseases around the world. These include prevalence-based definitions, “not more than” a fixed number of persons or a nationally defined list, as well as several qualitative descriptors. In the United States, the 1983 Orphan Drug Act defines a rare disease as a condition that affects fewer than 200,000 individuals in the population. Analogous legislation in the European Union, entered into force in 2000, defines a disease as rare when it affects fewer or equal to 5 in 10,000 persons [12]. China maintains a national list of rare diseases [13]. Less than a third of countries and areas in the world have legislation or regulation in place to identify a rare disease [4]. Of those countries, most recognize the threshold of 5 in 10,000 persons. It is necessary to recognizes that the frequency of some rare diseases, such as rare cancers and rare infectious diseases, are more precisely defined by incidence. In many countries, rare cancers are defined as malignancies when incidence is less than 6 individuals per 100,000 in a year [14]. A large proportion of rare diseases are congenital disorders, highlighting the importance to also consider incidence at birth and birth prevalence rates. Using prevalence to describe rare diseases is preferred as it is not affected by general growth in population size of a country.

The International Classification of Diseases (ICD) defines disease as ‘a set of dysfunction(s) in any of the body systems’, or a ‘known pattern of signs, symptoms, and findings’. Recognition of medical conditions based on a distinct clinical presentation is practical and suitable for rare conditions given the uneven diagnostic capabilities throughout the world, and because there are often limited data to describe the underlying cause of a rare disease. Setting a level of clinical severity to qualify a disease is not recommended, as this may unintentionally exclude conditions that deserve attention. Some medical conditions may be extremely rare, such that there are insufficient data and medical knowledge to diagnose the patient. Consequently, these medical conditions have not yet been determined or recognized by a name. Conditions that remain undetermined following full clinical investigation with all available scientific capabilities are rare diseases. Recent advances in precision medicine permits the classification of common disorders into smaller biomarker-defined subsets [15]. However, these subsets are not identifiable by clinical phenotypes or as distinct diseases.

**Table 1:** The essential elements and concepts of ‘disease’ and ‘rare’ incorporated in the core definition statement

*Definition of Rare*
− Recognizing all PLWRD − Using a population prevalence to characterize rarity − A global description while recognizing variability in geographical and country contexts − Recognizing other epidemiological thresholds may be better suited to some subgroups of rare conditions
*Definition of Disease*
− Based on a distinct clinical presentation or phenotype, aligning with the ICD − Inclusive of all rare conditions, irrespective of clinical severity − Inclusive of all rare conditions, irrespective of disease etiology − Inclusive of conditions that remain undetermined − Recognizing subgroups of rare diseases

### Core definition of rare diseases

The core definition is the foundation for the Operational Description of Rare Diseases. Take into consideration the concepts underlying rarity and disease, core definition of rare disease statement reads:

*“Persons Living With a Rare Disease face distinct and significant challenges that arise from the infrequency of their medical condition*, *such as a long diagnostic journey*, *inadequate clinical management*, *and limited access to knowledge and effective treatments. The burden of rare diseases on patients*, *their caregivers and families*, *healthcare systems*, *and society overall*, *merits greater visibility and recognition.*

*A rare disease is a medical condition with a specific pattern of clinical signs*, *symptoms*, *and findings that affects fewer than or equal to 1 in 2000 persons living in any World Health Organization-defined region of the world.*

*Rare diseases include*, *but are not limited to*, *rare genetic diseases*, *rare cancers*, *rare infectious diseases*, *rare poisonings*, *rare immune-related diseases*, *rare idiopathic diseases*, *and rare undetermined conditions. While the frequency of most rare diseases can be described by prevalence*, *some rare diseases*, *such as rare cancers and rare infectious diseases*, *can be more precisely described by incidence.”*

### Descriptive framework of rare diseases

Identifying PLWRD is key to improving their visibility in health systems and to allow for a baseline to monitor measurable change. To make the core definition operational, inform and guide new actions and policies, it must provide context. The Operational Description of Rare Diseases is thus framed in two parts: a core definition, complemented by a series of three descriptive frameworks. Both are indispensable to permit operational, practical implementation by a wide range of decision-makers who contribute to improve diagnosis, disease management, health and social equity outcomes for PLWRD.

#### Framework 1. PLWRD faces distinct challenges that arise from the infrequency of their medical conditions

Rarity itself is the source of many of the challenges that impact the rare disease population. There is a limited understanding of medical conditions with low frequency in the general population, primarily because there are only a small number of affected individuals. Clinicians across the world encounter these patients infrequently, which may compromise effective diagnosis, management, and treatment.

PLWRD navigate an uncertain diagnostic journey because clinicians and healthcare systems lack awareness and experience to recognize rare conditions [16, 17]. Patients remain undiagnosed for a long time, or may experience misdiagnoses, resulting in inappropriate treatment or irreversible disease progression. Even with a diagnosis, the clinical management of patients is often inadequate. Without sufficient knowledge about a rare disease, clinicians cannot assess its complexity, anticipate the disease course, or standardize health management practices [18]. Clinicians may be uncertain if an effective treatment exists. Geographic and economic barriers to care prevent patients from being directed along appropriate referral pathways to reach centers of expertise that may have the specialization to manage and treat the rare condition. The coordination across multidisciplinary health and social services is often inadequate. The transition from pediatric to adult care is also often poorly organized [19, 20]. In the absence of a global network for sharing the existing limited knowledge, these challenges may be more pronounced in low- and middle-resource countries and in indigenous populations [11, 21].

Effective treatment options are limited because research and development may not be prioritized and economic incentives may be insufficient [22–24]. Conducting small cohort trials across different countries poses methodological and ethical challenges [8, 25, 26]. Ultimately, if a treatment is developed, high pricing often results in delayed and inequitable access.

#### Framework 2. The burden of rare diseases merits greater visibility and recognition

A distinct rare disease affects a small number of individuals. However, considering the 6,000 to 8,000 rare diseases collectively, a sizeable portion of the general population (estimated 3–8%), or 300 million persons globally, are affected [3, 5]. Recognizing the breadth of rare diseases across the world and their impact on physical and mental health means that health and social care systems are key to prioritizing services and improve outcomes [27].

Many rare diseases are chronic, progressive conditions that result in debilitating impairments. Many are serious and life-threatening. Rare conditions are often described as *complex and multisystemic* because they may affect multiple organs and bring about comorbidities. Beyond the physical impact, PLWRD experience psychosocial consequences such as isolation, stigmatization, and discrimination.

The burden of rare diseases extends to families and caregivers, and impacts society at large [28, 29]. Approximately 70–90% of rare diseases are childhood-onset [5]. Parents, particularly mothers, assume caregiver responsibilities that have negative consequences to their physical, psychosocial, and financial well-being [28, 30]. Care responsibilities can hinder their full participation in other aspects of life, limiting the potential for educational and professional development of the caregivers thus generating isolation, exclusion, and impoverishment. Workplace productivity loss for PLWRD and caregivers significantly contributes to the economic burden of rare diseases [28, 31, 32].

#### Framework 3. The Operational Description of Rare Diseases is a catalyst for action

This global reference encourages clearer representation of rare diseases within the ICD, and the ‘International Classification of Functioning, Disabilities, and Health’, and its alignment with domain-specific terminologies. Improving the codification and classification of rare diseases at a global level will enhance the interoperability of data, and the alignment of reference terminologies across different health information systems. A common point of reference will facilitate international research efforts to understand the etiology, pathogenesis, and natural history of rare diseases. A global network of shared knowledge will facilitate research collaborations, foster innovation for new treatments, and highlight the need for equitable care services across different geographies, cultures, and economies [33].

Achieving Universal Health Coverage requires that the specific needs of PLWRD are addressed, “leaving no one behind” [1]. An improved understanding of the unmet needs of the rare disease population will encourage the integration of new strategies and services into medical care. For example, greater recognition of the challenges diagnosing a rare disease may promote the development of diagnostic criteria, or the expansion of testing, such as newborn screening programs. Other actions to improve access to care include the creation of national healthcare provider service planning and awareness campaigns in primary healthcare, or the development of referral pathways to facilitate access to local, national, or international centers of expertise. Recognition of the severity and complexity of many rare diseases may call for services to support the need for multi-disciplinary care, to improve both physical and mental health. Ultimately, adoption of this reference is essential to improve health outcomes and facilitate the holistic inclusion of PLWRD and their caregivers in society.

### Future considerations

Adoption of this Operational Description of Rare Diseases as a common reference is an essential step toward improving the recognition of all rare conditions and stimulate actions. The expert group recognized that adopting an international reference would have wide ranging intended or unintended consequences in terms of policy, incentives, provision of services and resources. It offers an opportunity for countries currently without a rare disease definitions to adopt this reference. However, countries with an existing definition may be resistant to change their interpretation of a rare disease. Nevertheless, the Operational Description of Rare Diseases supports a convergence towards a common understanding of rare diseases, by providing a baseline to for future discussions. Additional impact assessment should be carried out to better analyze the perceived challenges and opportunities to adopting a global reference.

## Conclusion

The Operational Description of Rare Diseases establishes a common point of reference to promote the convergence toward an international understanding of rare diseases. Adoption of the Operational Description of Rare Diseases as a global reference is an essential step toward improving the recognition and visibility of all rare conditions in health systems and will serve as a baseline to monitor measurable change at local, national, and international levels. It provides impetus to the calls within the 2019 UN Political Declaration on Universal Health Coverage and the 2021 UN Resolution “Addressing the Challenges of Persons Living with a Rare Disease and their Families”. By highlighting the distinct challenges faced by PLWRD, this reference will motivats new actions and policies to address the challenges faced by the global rare disease community.

## Electronic supplementary material

Below is the link to the electronic supplementary material.

Supplementary Material 1

## Data Availability

Not applicable.

## Declarations

### Authors’ information

AH is the Scientific Director of Online Mendelian Inheritance of Man (OMIM).

DA was the Chair of the Therapies Scientific Committee at IRDiRC.

FS is the Coordinator of the ERKNet, European Reference Network for Rare Kidney Disorders and President of the International Pediatric Nephrology Association.

HD was the Vice-Chair of IRDiRC, and was the Director of the Office of Population Health Genomics for the Department of Health, Government of Western Australia

GB was the chair of the Diagnostic Scientific Committee of IRDiRC and currently the chair of Interdisciplinary Scientific Committee of IRDiRC.

LM was the Chair of IRDiRC between 2019-2021.

## Abbreviations

PLWRD: Persons Living with a Rare Disease
ICD: International Classification of Diseases
UN: United Nations
WHO: World Health Organization
