# Supplementary material for: Operational description of rare diseases: a reference to improve the recognition and visibility of rare diseases
Source: Orphanet J Rare Dis. 2024 Sep 11;19:334. doi: 10.1186/s13023-024-03322-7 (PMC11389069; doi:10.1186/s13023-024-03322-7)
Supplement: Supplementary file 1 — Supplementary Material 1 [file 13023_2024_3322_MOESM1_ESM.docx]

**Supplementary Material**

**Methodology**

A multi-stakeholder group of 18 experts from six continents were invited by Rare Diseases International to take part in an Expert Group to develop the Operational Description of Rare Diseases. The participants included clinicians, researchers, terminology producers, medical statisticians, industry expert, patient representatives, policy makers and public health experts. The participants were selected based on their expertise profiles, geography, and organizations (e. g. IRDiRC, OMIM, WHO).

**Figure 1: Overview of the steps and process used to develop the Operational Description of Rare Diseases (image and modified-Delphi process adapted from Khodyakov *et al*., 2019).**


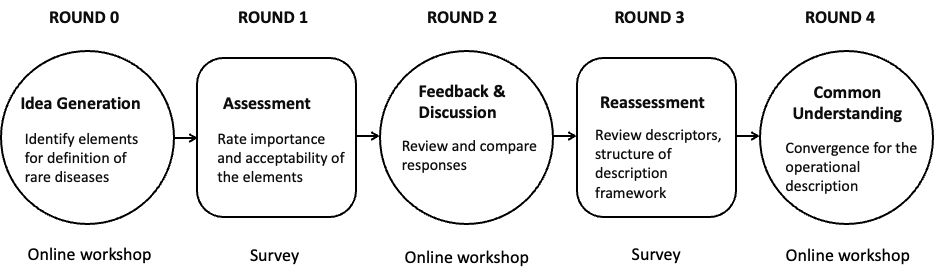


The online modified-Delphi process was used to build consensus (1). Experts participated in three online workshops and two surveys between November 2021 and January 2022 (Figure 1).

For the first workshop, experts provided statements on the essential elements for the definition of rare diseases*.* The key concepts were organized by Rare Diseases International staff into the first survey, containing thirty statements, which was then scored independently by the experts. Survey statements addressed: *objectives* and *elements* of an operational description (9 statements), *terminology (3 statements)*, *descriptors of ‘disease (5 statements)’*, ‘*rarity (7 statements)’*, and *qualitative descriptors (6 statements)*. Consensus was recognized if 70% of the responses were scored as strongly agree / agree. Discussion in the second workshop focused on topics where consensus was not reached, to work toward agreement on the structure and the essential elements for the operational description.

A second survey was developed that included twenty-five statements across the following areas: *objectives* and *structure* of an operational description*; Key elements of the core definition,* followed by statements specific to the core definition with a focus on *‘disease’,* and *‘rare’*. Considering the results of the two surveys and discussions during the workshops, an outline of the Operational Description of Rare Diseases was drafted by Rare Diseases International and presented during the third workshop. Discussion focused on seeking consensus on the descriptor of ‘rare’ and the final phrasing of the core definition. Additional reviewers from the community were invited to provide feedback on early drafts of the document; they are mentioned in the Acknowledgement. The final Operational Description of Rare Diseases was reviewed and refined by the Expert Group.

**Reference**

1. Khodyakov D, Denger B, Grant S, Kinnett K, Armstrong C, Martin A, et al. The RAND/PPMD Patient-Centeredness Method: a novel online approach to engaging patients and their representatives in guideline development. Eur J Pers Cent Healthc. 2019;7(3):470–5.
